# Platypnea-Orthodeoxia: An Effective Diagnostic Tool for Hepatopulmonary Syndrome With Chronic Obstructive Pulmonary Disease

**DOI:** 10.7759/cureus.35904

**Published:** 2023-03-08

**Authors:** Yuzo Furuta, Masataka Sugahara, Takahito Nakamura, Kazunari Tominaga, Yoshiyuki Kijima

**Affiliations:** 1 Department of Cardiology, Japan Community Healthcare Organization Hoshigaoka Medical Center, Osaka, JPN; 2 Department of General Internal Medicine, Nara Prefecture Seiwa Medical Center, Nara, JPN; 3 Department of Gastroenterology, Japan Community Healthcare Organization Hoshigaoka Medical Center, Osaka, JPN

**Keywords:** intrapulmonary arteriovenous shunt, microbubble contrast echocardiography, dyspnea, platypnea-orthodeoxia, hepatopulmonary syndrome

## Abstract

Hepatopulmonary syndrome (HPS) shows progressive dyspnea resulting from intrapulmonary atrioventricular shunts in liver cirrhosis. The comorbidity of chronic lung disease often hampers the diagnosis of progressive dyspnea in patients with HPS. Therefore, a comprehensive approach to the determination of dyspnea is required. Here, this case report shows that a patient with chronic obstructive pulmonary disease (COPD) and alcoholic liver cirrhosis was diagnosed with HPS after admission due to worsening dyspnea. Although COPD exacerbation was initially suspected because of the long history of smoking, physical examinations, laboratory findings, and imaging data, dyspnea remained after recovery from worsening respiratory failure. HPS was suspected due to the absence of increased CO_2_ levels and the presence of platypnea-orthodeoxia. We diagnosed the intrapulmonary arteriovenous shunt with microbubble-contrast echocardiography and technetium-99m macroaggregated albumin scintigraphy. Therefore, this case highlighted that HPS rather than COPD was suspected of hypoxemia associated with repositioning for the differential diagnosis of dyspnea.

## Introduction

Dyspnea results from multiple factors, including cardiac, respiratory, metabolic, musculoskeletal, and psychiatric factors. It is important to clarify the critical pathogenesis related to appropriate treatments for dyspnea. Hepatopulmonary syndrome (HPS) showing intractable dyspnea results from arterial hypoxemia due to a diffuse intrapulmonary arteriovenous shunt (expressing vascular dilatation), especially in the lower pulmonary lobes of patients with chronic hepatic diseases such as liver cirrhosis. HPS is characterized by the triad of chronic liver disease, arterial hypoxemia, and intrapulmonary arteriovenous shunting [1,2]. In addition, HPS is a progressive disease, and its prognosis is poor without liver transplantation [3]. We must pay attention to the possibility of HPS in patients with liver cirrhosis. We report a case of dyspnea with hypoxemia with liver cirrhosis and chronic obstructive pulmonary disease (COPD) diagnosed with HPS on the basis of poor oxygenation caused by a position change from supine to sitting.

## Case presentation

A 55-year-old man with alcoholic liver cirrhosis (Child-Pugh classification C) was managed by a hepatologist for the manifestation of worsening hypoxemia. Saturation of pulse oximetry oxygen (SpO2) was always checked, as the patient’s daily routine because he had COPD with ongoing smoking of 10-40 cigarettes/day for 35 years. Usually, the SpO2 of the patient was 92% on room air. However, on the day before admission, dyspnea worsened even with light exertion and SpO2 dropped to 69%. On admission, SpO2 was 87% without oxygen supply in the sitting position but rapidly recovered to 96-98% with 2 L/minute of oxygen. Then, the oxygen supply could be reduced to 1 L/minute in the supine position. Pulmonary sounds were bilaterally auscultated mid to late expiratory wheezes. Heart sounds were normal. In addition, the patient had yellow ocular conjunctiva, splenomegaly, digital clubbing, gynecomastia, and pitting edema of the bilateral lower legs.

**Figure 1:**
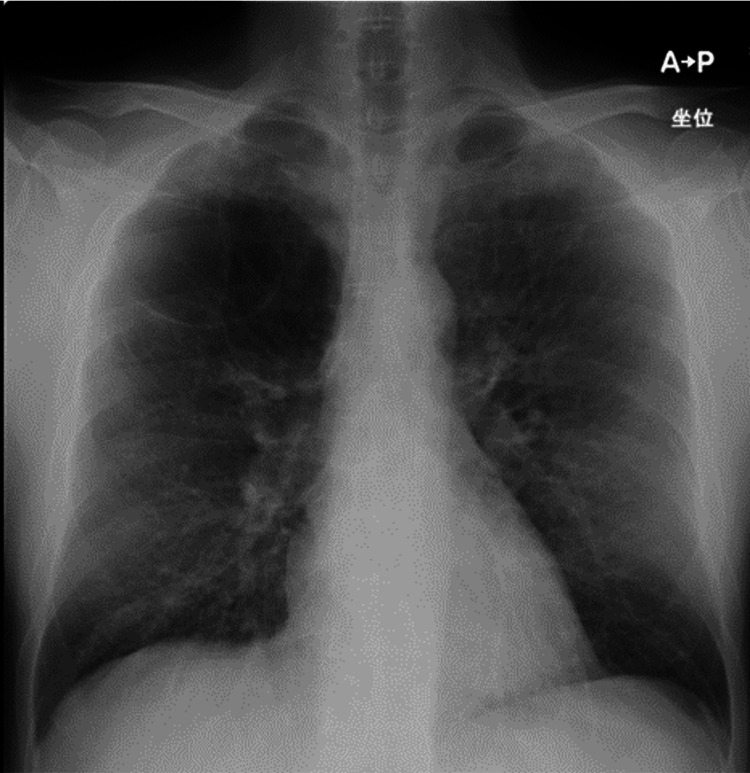
Chest X-ray on admission showed hyperinflation of both lungs

**Figure 2:**
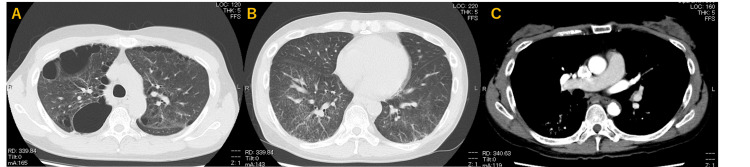
Plain CT showed a bulla at the periphery of the bilateral upper lobes (A), and ground-glass opacities extending to both lobes (B). Contrast-enhanced CT showed no enlargement of the pulmonary arteries or thrombus in the pulmonary arteries (C).

Arterial blood gas data without oxygen supply at rest showed partial pressure of carbon dioxide (PCO2) was not high and the first alveolar-arterial oxygen gradient (AaDO2) was relatively large (Table 1). Laboratory data showed that liver function was low and N-terminal pro-brain natriuretic peptide, an indicator of heart failure, was within reference values. Chest X-ray showed hyperinflation of both lungs (Figure 1). Plain CT showed a bulla at the periphery of the bilateral upper lobes (Figure 2A), and ground-glass opacities extending to both lobes (Figure 2B). Contrast-enhanced CT showed no enlargement of the pulmonary arteries or thrombus in the pulmonary arteries (Figure 2C). Transthoracic echocardiography showed normal biventricular function without pulmonary hypertension (tricuspid regurgitant velocity was 2.1 m/sec) and valvular disease.

**Table 1:** Arterial blood gas data without oxygen supply at rest showed PCO2 was not high and AaDO2 was relatively large. Laboratory data showed decreased liver function. AADO2: airst alveolar-arterial oxygen gradient; PCO2: partial pressure of carbon dioxide

Laboratory test	Measured values	Reference
Arterial blood gas		
pH	7.452	7.35-7.45
partial pressure arterial carbon dioxide (mmHg)	32	35-45
partial pressure arterial oxygen (mmHg)	52	> 75
bicarbonate (mmol/L)	21.8	22-26
alveolar-arterial gradient (mmHg)	53	< 10
Blood examination		
albumin (g/dL)	2.8	4.1-5.1
total bilirubin (mg/dL)	6.0	0.2-1.2
aspartate aminotransferase (IU/L)	30	< 40
alanine aminotransferase (IU/L)	14	< 40
creatinine (mg/dL)	0.77	0.46-0.79
C-reactive protein (mg/dL)	1.60	< 0.30
N-terminal pro-brain natriuretic peptide (pg/mL)	31	0-125
prothrombin time activity (%)	34	70-120
Complete blood count		
white blood cell count (/µL)	7800	3600-9000
red blood cell count (*10^4^/µL)	379	387-525
platelet count (*10^4^/µL)	5.6	13.8-30.9

He received oxygen supply (1-2 L/min) and ceftriaxone (1 g once daily) for six days. Despite the inflammatory indices having recovered, the patient’s physical activity remained low because of worsening dyspnea on exertion.

**Figure 3:**
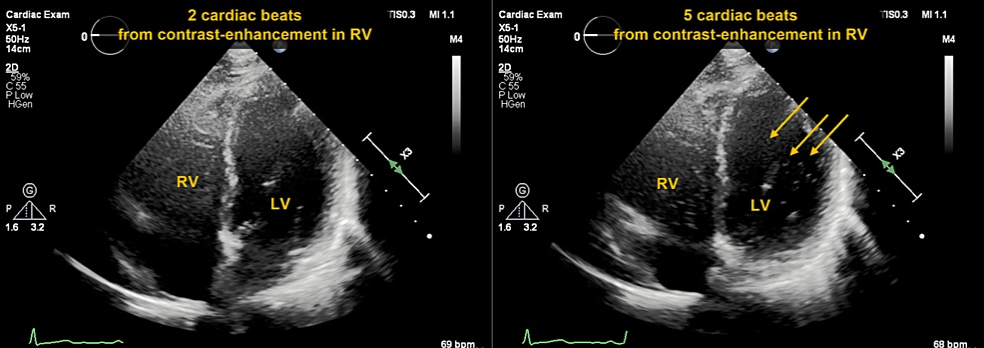
Microbubble-contrasted echocardiography from the apical four-chamber view showed opacifications (arrows) in the left ventricle after five beats from entering the right ventricle.

**Figure 4:**
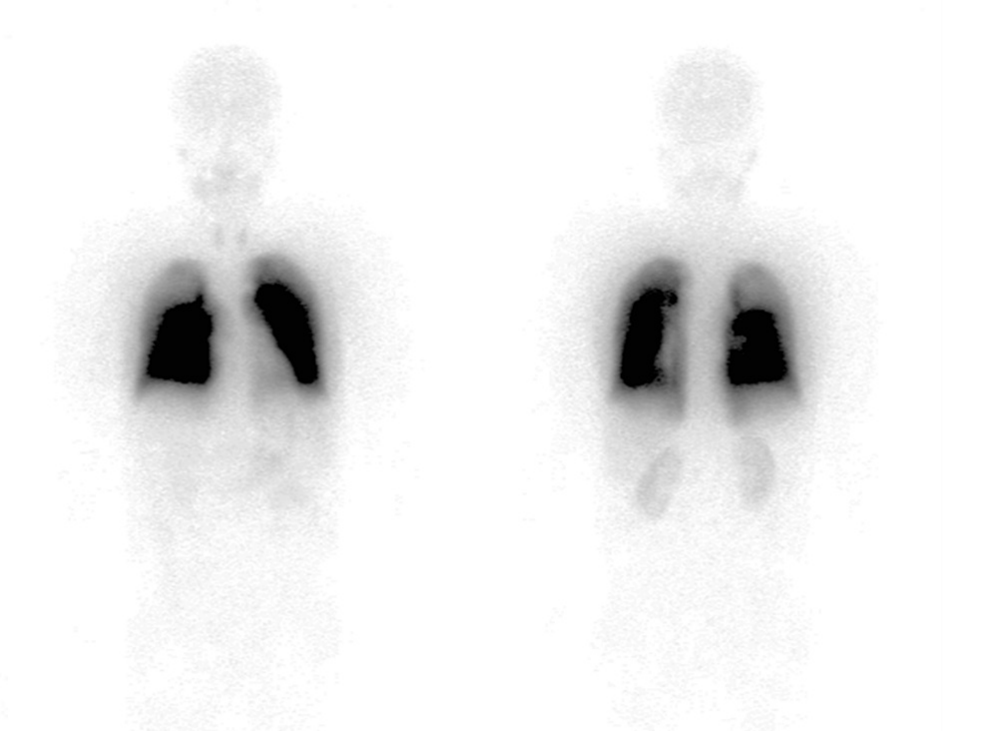
Lung perfusion scintigraphy with technetium-99m macroaggregated albumin (TcMAA) Radioactivity was measured from the anterior (left) and posterior directions (right) in two different regions of interest, both lungs (L) and whole body (WB). The shunt ratio was calculated to be 11.4% (anterior) and 9.2% (posterior) (mean value 10.3%).

After recovery from worsening respiratory failure with antimicrobial therapy, the pulmonary function test showed 63.2% of forced expiratory volume in one second, 84.4% of forced vital capacity, and 42.4% of diffusing capacity of the lung for carbon monoxide. The six-minute walk distance was 360 m (68% of the expected distance). During the walk, SpO2 decreased from 95% at the beginning to 88% at the end without oxygen supply. In addition, we found a decrease in SpO2 in the upright position (96% in the supine position to 91% in the upright position). Microbubble contrast echocardiography (CE) was performed to confirm the intrapulmonary arteriovenous shunt. We found opacifications in the left ventricle at a five-cardiac cycle delay from opacification of the right ventricle (Figure 3). A technetium-99m macroaggregated albumin (TcMAA) examination also showed 10.3% of a shunt fraction and no evidence of defects of lung perfusion and lung thromboembolism (Figure 4).

All these findings indicated an abnormal increase in shunt flow, which led to the diagnosis of HPS. The patient rejected liver transplantation and hoped for conservative treatments, including oxygen therapy and respiratory rehabilitation for the improvement of hypoxic stress conditions.

## Discussion

For the differential diagnosis of dyspnea, the frequency of the disease in medical practice and the age, symptoms, and signs of patients can be helpful. Although orthopnea associated with left heart failure is one of the most common types of dyspnea, worse dyspnea and hypoxemia in the upright position compared with the supine position appeared in this case (so-called platypnea-orthodeoxia syndrome). This finding must be helpful in the diagnosis of HPS.

We initially suspected COPD exacerbation because of the long history of smoking, physical examination, such as bilaterally auscultated mid to late expiratory wheezes, and chest imaging findings. However, PCO2 was not increased (32 mmHg), which was inconsistent with the present pathological conditions. In addition, AaDO2 was relatively large, suggesting the possibility of combination with multiple pathogeneses such as shunt disease, poor diffusion capacity, and unequal ventilation blood flow. Since the respiratory conditions could not be explained by COPD exacerbation alone, we focused on platypnea-orthodeoxia and suspected HPS as a complication. Diagnostic criteria for HPS are shown in Table 2 [1,2].

**Table 2:** Diagnostic criteria of HPS AaDO2: alveolar-arterial oxygen gradient; 99mTc-MAA: technetium-99m macroaggregated albumin

The following criteria should be fulfilled.
1. Presence of liver disease and/or portal hypertension
2. PaO_2_ < 80 mmHg or AaDO_2_ ≥ 15 mmHg in room air
3. Intrapulmonary vascular dilatation by contrast echocardiography or 99mTc-MAA

Although COPD often causes shortness of breath and hypoxemia on exertion, HPS has also been reported to occur in patients with liver cirrhosis, causing hypoxemia on exertion [4]. Based on the physical findings of platypnea-orthodeoxia, we were able to diagnose HPS complicated with COPD. There has been a previous report of HPS and COPD complications [5], and HPS should be diagnosed because of its poor prognosis.

HPS is characterized by the redistribution of blood flow to the shunt and exacerbation of hypoxemia due to pulmonary vasodilation mainly at the lung base [6]. Such vascular changes are caused by increased production of both nitric oxide in vascular endothelial cells and vascular endothelial growth factor-A from intravascular macrophages [7]. The diagnostic criteria for HPS are shown in Table 1. CE or TcMAA is useful for clarification of intrapulmonary shunts. CE is performed by the infusion of saline solution with a small amount of air from a peripheral vein. In normal subjects, microbubbles can be trapped in 8-15 µm capillaries in the lungs and cannot appear in the left ventricle. On the other hand, the appearance of microbubbles in the left ventricle is strongly suspected to have intra- or extra-cardiac shunts in this case. When opacification appears in the left ventricle after more than three cardiac cycles from entering the right ventricle, it is highly suspected that the patient has an intrapulmonary shunt (extracardiac shunt), but not an intracardiac shunt [8]. Although pulmonary blood flow scintigraphy shows the accumulation of TcMAA in other organs due to intrapulmonary shunt, CE can more directly diagnose the presence of right-left shunt than TcMAA.

Patients with HPS had a higher model for end-stage liver disease (MELD) scores (this score is used as a criterion to predict 3-month survival and prioritize liver transplantation in patients with liver cirrhosis), significantly higher prevalence of spider angiomas, clubbed fingers, dyspnea, and platypnea than patients without HPS [9]. Therefore, we can suspect HPS by the presence of these symptoms, and measuring oxygenation in the sitting and supine positions can help in the diagnosis. We have noticed the existence of platypnea-orthodeoxia syndrome, which leads to the diagnosis of HPS based on the patient's background. CE is a useful and non-invasive method for HPS diagnosis, particularly in patients with liver cirrhosis and chronic pulmonary diseases. Although this patient had pulmonary disease, it was important for the precise diagnosis to recognize the comprehensive physical findings that the cause of hypoxemia is not only pulmonary disease but also other factors.

## Conclusions

We experienced a case of HPS diagnosed by CE as a cause of worsening dyspnea on exertion and hypoxemia due to both chronic respiratory disease and liver cirrhosis. Although the differential diagnosis of dyspnea is broad, we should consider the presence of HPS in poor oxygenation associated with repositioning in cases concomitant with liver cirrhosis.
